# Supplementary material for: A set of multi-entry identification keys to African frugivorous flies (Diptera, Tephritidae)
Source: Zookeys. 2014 Jul 24;(428):97–108. doi: 10.3897/zookeys.428.7366 (PMC4143993; doi:10.3897/zookeys.428.7366)
Supplement: Supplementary material 6 — Key to Ceratitis [file zookeys-428-097-s006.zip › SF6_ZooKeys_key to Ceratitis/key/SF6_ZooKeys_key to Ceratitis/Media/Html/Ceratitis edwardsi.htm]

Ceratitis (Pardalaspis) edwardsi (Munro) 


***Ceratitis*** **(*Pardalaspis*) *edwardsi*
(Munro)**

*Pardalaspis* *edwardsi* Munro, 1957: 868.

Body
length: 8.0 (7.5-8.5) mm; wing length: 8.08 (7.5-9.0) mm.

Male

Head.
Antennal segments yellow-orange. Third antennal segment three times as long as
second segment. Arista dark over entire length. Frons yellow in ground
colour,with indistinct silvery sheen especially in centre; with dispersed short
hairs, distinctly darker than frons. Ocellar triangle dark. Face orange to dark
orange; no median band. Occiput moderately swollen below, pale whitish.
Chaetotaxy normal for subgenus.

Thorax.
Ground colour of mesonotum brownish; with three darker longitudinal stripes,
central line broad, lateral ones interrupted and thinner, darker markings
around prescutellar acrostichal setae. Postpronotum same colour as mesonotum.
Chaetotaxy normal for subgenus. Mesonotum with mainly dark pilosity.
Anepisternum along upper margin and lower half with darker hairs. Two
anepisternal bristles.  Scutellum yellow with apical markings black; basally with
two dark spots.

Legs
yellow to orange-brown, setation typical of subgenus; predominantly dark.
Posterior row on front femur dark.

Wings
with brownish bands, yellow markings strongly reduced. Banding, setation and
venation normal for subgenus. Marginal band continuous; discal band joined with
marginal band; cross-vein r-m at middle of discal cell; vein R_1_
ending before cross-vein r-m.

Abdomen.
Pale greyish, with clearly defined spots. Pattern of spots and setation normal
for subgenus.

Female

As
male except for the following characters. Face and frons variable, from pale
yellow to yellow-orange. Mesonotum with pale pilosity except along longitudinal
stripes where there are dark hairs. Cross-vein r-m before middle of discal
cell; R_1_ ending before or opposite cross-vein r-m. Abdomen with
orange tinge. Oviscape moderately long, at least as long as abdominal terga
3-6; orange in colour.  

(Description
after De Meyer, 1996)
